# Population Receptive Field Characteristics in the between- and Within-Digit Dimensions of the Undominant Hand in the Primary Somatosensory Cortex

**DOI:** 10.1093/cercor/bhab097

**Published:** 2021-05-10

**Authors:** Luyao Wang, Zhilin Zhang, Tomohisa Okada, Chunlin Li, Duanduan Chen, Shintaro Funahashi, Jinglong Wu, Tianyi Yan

**Affiliations:** 1 School of Mechatronical Engineering, Beijing Institute of Technology, Beijing 100081, China; 2 Department of Psychiatry, Graduate School of Medicine, Kyoto University, Kyoto 606-8507, Japan; 3 Human Brain Research Center, Graduate School of Medicine, Kyoto University, Kyoto 606-8501, Japan; 4 School of Biomedical Engineering, Capital Medical University, Beijing 100069, China; 5 School of Life Science, Beijing Institute of Technology, Beijing 100081, China; 6 Advanced research institute of multidisciplinary science, Beijing Institute of Technology, Beijing 100081, China

**Keywords:** high-resolution fMRI, population receptive field, primary somatosensory cortex, somatotopic map, tactile

## Abstract

Somatotopy is an important guiding principle for sensory fiber organization in the primary somatosensory cortex (S1), which reflects tactile information processing and is associated with disease-related reorganization. However, it is difficult to measure the neuronal encoding scheme in S1 in vivo in normal participants. Here, we investigated the somatotopic map of the undominant hand using a Bayesian population receptive field (pRF) model. The model was established in hand space with between- and within-digit dimensions. In the between-digit dimension, orderly representation was found, which had low variability across participants. The pRF shape tended to be elliptical for digits with high spatial acuity, for which the long axis was along the within-digit dimension. In addition, the pRF width showed different change trends in the 2 dimensions across digits. These results provide new insights into the neural mechanisms in S1, allowing for in-depth investigation of somatosensory information processing and disease-related reorganization.

## Introduction

Tactile information needs to be transmitted from peripheral mechanoreceptors through the spinal cord, dorsal column nuclei, and ventral posterolateral nucleus (VPL) of the thalamus to the somatosensory cortex. Neuronal responses are topologically processed tactile information along this pathway and ultimately project in the primary somatosensory cortex (S1), which is called somatotopic organization. This is one of the important neuronal encoding schemes in S1, which was first drawn by Penfield as a homunculus (Penfield and Boldrey 1937; Penfield and Jasper 1954; Schott 1993). Researchers applied intraoperative electrical cortical stimulation in the pre- and postcentral gyri on epileptics and observed or asked participants which part of the body was experiencing the feeling. With the development of magnetic resonance imaging (fMRI), many studies have focused on measuring somatotopic maps of S1 by noninvasive methods. When stimulating certain body parts, the corresponding activation area of the brain can be detected (Zeharia et al. 2015). Tactile information on different body parts is projected to S1 in a certain order (Abraira and Ginty 2013). According to the homunculus drawn by Penfield, there is an inverted body map lined up over the cortical surface. The reorganization of this somatotopic map is always accompanied by pathological processing, such as in patients with dystonia (Mantel et al. 2016), carpal tunnel syndrome (Maeda et al. 2014), cerebral palsy (Papadelis et al. 2018), and spinal cord injury (Saadon-Grosman et al. 2015). A study found that reorganization occurred even after 24 h of gluing manipulation, which influenced the discrimination performance (Kolasinski et al. 2016a). Investigating the somatotopic map is important for understanding and characterizing the tactile processing pathway and related diseases (Wesselink et al. 2019).

Studies on 2-point discrimination thresholds have shown that the glabrous skin of the hand is the area of highest spatial acuity (Mancini et al. 2014; Zhang et al. 2018). To recognize an object, it is critical to know which parts or fingers of our hand are in contact with the object, meaning that there is a correspondence relationship between the external stimulus on each digit and S1 representations. Numerous studies have confirmed that hands and faces occupy the largest areas in S1. Using ultrahigh field (7 T) fMRI, it is possible to research the brain’s responses to stimulation of individual digits (Besle et al. 2013). The somatotopic organization of the hand has been established in S1, with the representation of the thumb being the most inferior and lateral, and other digits are represented at increasingly superior and medial locations followed by the palm (Sanchez-Panchuelo et al. 2010). In general, the arrangement of the brain activation region corresponds to anatomical structure in between-digit dimension. These findings are consistent with nonhuman primate studies (Paul et al. 1972; Kaas et al. 1979). In addition, some studies found multiple hand representations that were related to within-digit dimension maps (proximal to the distal phalanx) orthogonal to the central sulcus (Schweisfurth et al. 2011; Sanchez-Panchuelo et al. 2012; Schweisfurth et al. 2014). However, most previous studies only focused on the right hand because it is the dominant hand in most people’s daily lives. The somatotopic organization of the left hand has not been investigated clearly, hindering our full understanding of tactile information processing in the brain.

When a digit contacts an object, a specific neuron population is activated. Invasive monkey studies found that the neuronal response characteristics of the somatosensory cortex were similar to those of mechanoreceptors on the skin (Delhaye et al. 2018). However, it is difficult to measure the response properties in awake and behaving humans. Previous studies only focused on cortical magnification, which reflects the relationship between tactile stimuli and activation strength or area in S1. This ignored the interaction between stimulus and the neuron receptive field, such as the “on” and “off” zones (i.e., excitatory and inhibitory surroundings of neurons) (DiCarlo and Johnson 2000). Population receptive field (pRF) mapping in 1 solution has been widely used in retinotopic mapping at the fMRI level (Dumoulin and Wandell 2008; Zuiderbaan et al. 2012). This method models a population of cells contained within a single fMRI voxel. Studies have confirmed the consistency of pRF size measured by fMRI and single neurons (Keliris et al. 2019). To the best of our knowledge, only 1 study has used a 1D Bayesian model to preliminarily explore the pRF characteristics in S1 (Puckett et al. 2020). However, the human hand could be modeled into a 2D space (e.g., between-digit and within-digit dimensions). They only focused on the 4 digits of the right hand in the between-digit dimension in total S1, which ignored the pRF configuration in the within-digit dimension. In addition, a previous study reported consistent ordering across participants for small but not index digits on the right hand, which confirmed that the somatotopic map was highly usage-dependent (Schweisfurth et al. 2015). Studying the undominant hand could reveal the general pRF characteristics of humans in S1.

To investigate this, we established a 2D hand space and designed a phase-encoding experiment to obtain S1 activation in a 7 T-fMRI environment. We recruited right-handed participants and focused on the activation of their left hand, including the 5 digits and the palm. The pRF was measured in between- and within-digit dimensions using Gaussian and difference of Gaussian (DoG) ellipse models. In addition, based on Brodmann’s cytoarchitectural studies and our functional results, the primary S1 cortex was divided into 4 separate subareas (BA3a, BA3b, BA1, and BA2). Previous studies found that somatosensory information is processed hierarchically in subareas of S1 (e.g., BA3a receives proprioceptive information, BA3b and BA1 process signals from skin, and BA2 combines the signals) (Costanzo and Gardner 1980; Gardner 1988). We hypothesize that the somatotopic map of the left hand is also represented in a certain order with low variability across participants. In addition, the pRF had different properties in between- and within-digit dimensions across digits and subareas.

## Materials and Methods

### Participants

Ten participants took part in the experiments (8 females, 24–31 years, mean 26.8 years). All of them were right-handed according to the Edinburgh Handedness Inventory (mean 87, SD 9.798) (Oldfield 1971). All the participants were healthy with normal visual, auditory, and tactile senses and had no history of neurological or psychiatric dysfunction. Data from 1 participant were excluded because of excessive head movement. This yielded a total of 9 participants with complete analyses. The protocol and data collection of the study were approved by the ethics committee of Kyoto University in accordance with the Declaration of Helsinki. Written informed consent was obtained from each participant following a detailed explanation of the study.

### Stimulation and Tasks

Tactile stimuli were presented using a pneumatic air-jet stimulator system, which could provide steady stimulus to the hand (Jia et al. 2020). The pressure of tactile stimuli applied to the fingers and palm was controlled at 150 mN by adjusting the input air pressure. The palm, proximal and distal phalanges of the thumb (D1), index (D2), middle (D3), ring (D4), and small (D5) digits of the left hand were stimulated using a phase-encoding design (Sanchez-Panchuelo et al. 2010; Sanchez-Panchuelo et al. 2012; Kolasinski et al. 2016b). For this, the experiment was divided into 2 orthogonal stimulus dimensions: a between-digit dimension (D1-D2-D3-D4-D5-Palm) to generate phase-related activity across the somatotopic representation of the fingers and palm in S1 and a within-digit dimension: distal phalanx (P1), proximal phalanx (P2), and palm.

**Figure 1:**
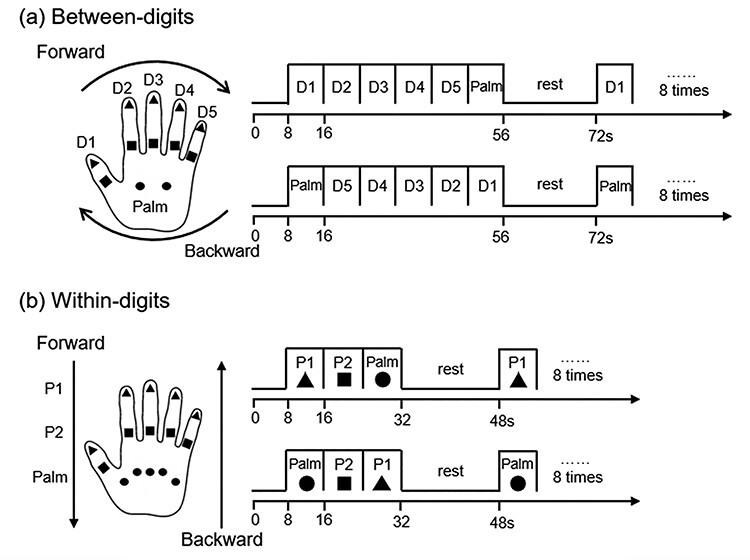
Phase encoding experimental design. (*a*) Between-digits session. Points with the same color were stimulated together. There are2 directions: forward (D1-D2-D3-D4-D5-Palm) and backward (Palm-D5-D4-D3-D2-D1). (*b*) Within-digits session. There are also 2 directions: forward (P1-P2-Palm) and backward (Palm-P2-P1). All 5 phalanges with the same shape were stimulated together.

For 1 cycle of the between-digit paradigm, stimuli were applied sequentially beginning with the thumb finger to the little finger and ending with the palm with no rest (6 locations in total, D1, D2, D3, D4, D5, and Palm). To control the stimulus input in each dimension, 2 points were stimulated simultaneously (points with the same color in Fig. 1*a*) in the between-digit dimension. Five points (points with the same shape in Fig. 1*b*) were stimulated simultaneously in 1 cycle of the within-digit paradigm, including distal (P1), proximal phalanges (P2) and palm. Each location was stimulated for 8 s before moving to the next location. Based on our previous study (Wang et al. 2020), the stimulus parameter was selected to ensure optimal brain activation (1 Hz stimulation with 700 ms continuous duration and 300 ms gap). Stimuli were delivered in either a forward (D1-D2-D3-D4-D5-Palm, P1-P2-Palm) or backward order (Palm-D5-D4-D3-D2-D1, Palm-P2-P1) to reduce the influence of hemodynamic response delay. Each run consisted of 8 cycles of stimulation. To control the attentional state, participants were asked to count the number of trials in each run.

### MRI Data Acquisition

**Figure 2:**
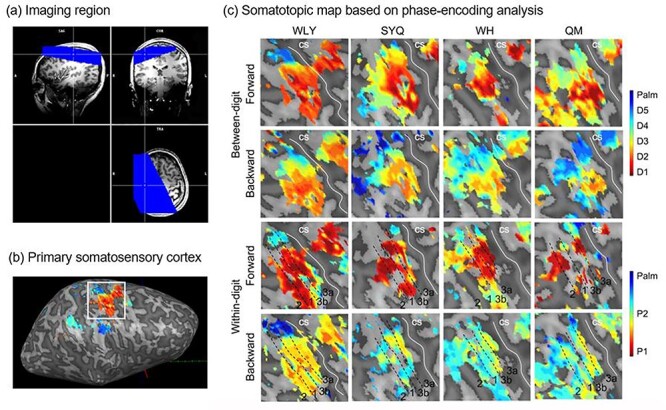
Somatotopic map based on phase-encoding analysis. (*a*) A total of 24 slices are tilted to cover the (*b*) hand area in the right hemisphere. (*c*) Activations of forward and backward order are shown independently. The top 2 rows are the results of the between-digit dimension, which show the representation of the hand with a certain order (e.g., from D1-D2-D3-D4-D5-Palm, red to blue). The bottom rows are the results of the within-digit dimension, which show the mirror layout at the boundaries of Brodmann 3a, 3b, 1, and 2 (e.g., P1-P2-Palm, red to blue).

Data were acquired on a MAGNETOM 7 T whole-body research scanner (Siemens Healthcare, Erlangen, Germany) with a 32-channel head coil (Nova Medical, Wilmington, USA). Functional data were obtained using echo-planar imaging (EPI) with the following parameters: TR = 2000 ms, TE = 32 ms, flip angle = 60°, matrix size = 128 x 128, parallel imaging factor = 3. A total of 24 slices were tilted to cover the hand area in the right hemisphere with 1-mm isotropic resolution (Fig. 2*a*). Whole-brain high-resolution anatomical images were collected using an MP2RAGE sequence with TR = 6000 ms, TE = 2.9 ms, flip angle = 4°/5°, matrix size = 256 × 284 × 247, first inversion time (TI1) = 270 ms, second inversion time (TI2) = 800 ms, GRAPPA with R = 3 acceleration (32 reference lines), and isotropic voxel size = 0.7 mm.

### MR Preprocessing

Standard preprocessing of data was carried out using BrainVoyager QX software (version 2.6, Brain Innovation B.V., Maastricht, the Netherlands), including 3D motion correction (trilinear interpolation for detection and sinc for correction), slice scan time correction and high-pass filtering (cutoff: 2 cycles per scan), but no spatial smoothing was performed. Anatomical datasets were resampled to 0.4 × 0.4 × 0.4 mm^3^ resolution, preprocessed, and transformed to ACPC space. Functional and anatomical datasets for each participant were coregistered and normalized to standardized Talairach space. The automatic segmentation routine was used to reconstruct the cortical surface at the white–gray matter border (with hand editing to minimize segmentation errors), and the resulting smooth 3D surface was partially inflated. We only used surface data for visualization and region of interest (ROI) definition because the spatial relationships among surface vertices are more straightforward to interpret (Puckett et al. 2020).

### Phase-Encoding Analysis

Phase-encoding analyses were performed using BrainVoyager QX based on the lag function. A modeled time course was composed of a gamma-convolved boxcar, which responded to the first 2 s of each stimulus cycle (corresponding to the TR), with no response to the remainder of the stimulus cycle. Then, this boxcar was shifted successively in 2-second increments to generate a series of lagged functions. Linear correlation was applied to these lagged functions and measured fMRI time course. Each voxel was assigned to the lag value with the highest correlation with its time course (winner-take-all). Lag values were then separately averaged for forward and backward order. Only voxels with a correlation threshold above 0.25 were considered somatotopic and included for further analysis (Thomas et al. 2015; Kolasinski et al. 2016b).

Results were mapped to the surface to define ROIs. The borders of Brodmann 3a, 3b, 1, and 2 were identified based on probabilistic maps (Martuzzi et al. 2014) and within-digit mirrored representations (Sanchez-Panchuelo et al. 2014). Surface ROIs were then mapped back into the brain volume (anatomical space) and expanded to include voxels from −1 to 3 mm around the gray–white matter boundary (Thomas et al. 2015). Preprocessed time-course data for each 3D anatomical voxel within the volume ROI were then exported to MATLAB for pRF modeling. All modeling and statistical analyses were performed using volumetric data.

### Bayesian pRF Modeling

The pRF estimation was performed using the BayespRF Toolbox (available from https://github.com/pzeidman/BayespRF) and SPM12 (available from http://www.fil.ion.ucl.ac.uk/spm) in MATLAB R2018b (Zeidman et al. 2018; Puckett et al. 2020). This method considers the interaction of stimulus and pRF size and is not affected by the experimental design. Our experiment contains 2 orthogonal dimensions (e.g., between-digit and within-digit). For this reason, we defined a 2D hand space, which was limited to ±12 units in both dimensions based on a previous study (Puckett et al. 2020). Since the range of space was arbitrarily defined, we slightly expanded it from 20 to 24 because there were more stimulus locations included in our study. The space was defined in the Cartesian coordinate system, which was different from the polar coordinate system of visual space. The hand space was divided into 6 segments along the *x*-axis (between-digit dimension) and 3 segments along the *y*-axis (within-digit dimension) of equal width, which corresponded to the digits and phalanges. All voxels used the same prior with a size of half of the space range and positioned in the origin of the coordinates.

We used both the Gaussian ellipse model and the DoG ellipse model to estimate the pRF characteristics within ROIs. The Gaussian ellipse model was an excitatory distribution with 4 estimated parameters: the location of the pRF center (*x* and *y*) and the widths along the *x*- and *y*-axes (the deviation of the profile, ${\sigma}_x$ and ${\sigma}_y$). The DoG model was constructed as the difference between an excitatory surround and an inhibitory surround. It has an extra parameter: the difference between the widths of the 2 Gaussians$({\sigma}_d$). The location of the pRF center was estimated to be between ±12, which fell within the defined hand space. In addition, ${\sigma}_x$ and ${\sigma}_y$ were constrained between 0.5 to 24, whereas ${\sigma}_d$ was 0–24.

**Figure 3:**
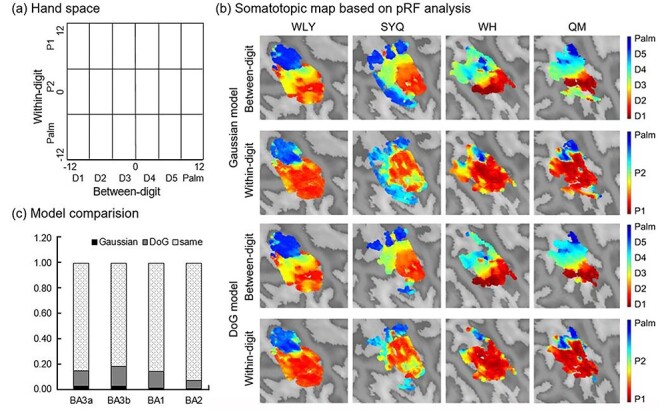
Somatotopic map based on Bayesian pRF analysis. (*a*) Hand space used in the modeling process. (*b*) Activation representations of the Gaussian and DoG models, which are similar to the results of the phase-encoding analysis. (*c*) Model comparison based on negative variational free energy.

After setting the parameters, voxel-wise analysis was performed by fitting the estimated waveform and measured BOLD time course extracted according to the phase-encoding analysis. The optimal pRF parameters were found by modifying the location and size of the model using the grid search method. In addition, we compared the performance of the Gaussian and DoG models based on an approximation of the model evidence—the negative variational free energy (F_Gaussian_ and F_DoG_). This is a sensitive approximation which could find the model with the most accurate and least complex explanation (Fig. 3*c*). For further analysis, we only used the results of DoG pRF because they combined the forward and backward sessions and could explain the activation of most voxels. Similar to the procedure of (Zeidman et al. 2018), a generative model was specified, in which the neuronal and hemodynamic parameters were estimated together. Neuronal activity was modeled using multivariate normal probability density functions, and hemodynamic response was modeled using the extended Balloon model. We used the default parameters set for 7 T data in the BayespRF Toolbox.

### Location and Extended Areas of the Hand

We calculated the variability of pRF center location in all ROIs across participants and digits based on the Dice coefficient (Eq. 1) (Dice 1945), which varied from 0 (no overlap) to 1 (perfect overlap).

(1) \begin{equation*} D=\frac{2\times \left|A\cap B\right|}{\left|A\right|+\left|B\right|} \end{equation*}

A 54 × 54 matrix was generated for all possible participant (*n* = 9) and digit pairings (*d* = 6). Furthermore, we measured the dominance ratio (MDR) based on the submatrix (Eq. 2), which compared the intradigit (diagonal) and interdigit (off-diagonal) overlap degrees across participants.

(2) \begin{equation*} M=\frac{\frac{1}{n}{\sum}_a{K}_{aa}}{\frac{1}{n\left(n-1\right)}{\sum}_{a,b,a\ne b}{K}_{ab}} \end{equation*}

The permutation test was applied to the matrix to quantify the likelihood of observing patterns and the statistical significance of the MDR value.

In addition, we extracted the location of the peak voxel with the highest F_DoG_ of each digit and palm. The peak voxel of thumb is regarded as the coordinate origin. The relative distance was calculated using Euclidean distance in 3D space between D1 and D2, D1 and D3, D1 and D4, D1 and D5, D1 and Palm. Because the total volume across participants was different, we analyzed the activation area using the relative volume to account for volumetric differences. The number of activated voxels of each digit and palm were normalized by the total number of voxels (Wang et al. 2020). In other word, the fraction occupied by a given digit or palm were measured.

### pRF Characteristics and Somatosensory Field Coverage

As we used an elliptical model to estimate the pRF characteristics, the width of the pRF along the between- and within-digit dimensions was recorded for each voxel. To investigate the relationship between pRF width and activation, we performed Pearson correlation analysis between the width of pRF and relative volume in two-dimensions independently (bootstrap resampling were conducted with 5000 samples) to examine their relationship. For the DoG model, the suppression index was used to indicate the center-surround configuration (Zuiderbaan et al. 2012). We took into account the total volume of the 2 Gaussians that make the pRF (Eq. 3).

(3) \begin{equation*} SI=\frac{\beta_i{x}_i{y}_i}{\beta_e{x}_e{y}_e} \end{equation*}

In addition, the distribution of pRF in each ROI were displayed to account for the primary center position and the spread distribution of pRF. We summed the receptive field across voxels in each ROI to form somatosensory coverage maps. We normalized the pRF characteristics for each participant. The maps were created individually and averaged together, which ranged from 0 to 1.

### Statistics

SPSS version 23 (SPSS, Inc., Chicago, IL) was used for statistical analysis. A paired-sample *t*-test was performed to test relative volume and pRF characteristics for all digit pairings (bootstrap resampling were conducted over subjects with 5000 samples). The significance level was set to α = 0.05.

## Results

### Somatotopic Map in S1

We first explored the somatotopic map based on phase-encoding analysis. The threshold (*r* > 0.25, *P* < 0.05, FDR corrected) activation representation displayed a clear and specific pattern in the right postcentral gyrus (Fig. 2). We displayed the results of forward and backward order independently. For the between-digit dimension, the location of the observed representation of the hand is arranged along the center sulcus in a certain order: thumb (D1), index (D2), middle (D3), ring (D4), and small (D5) digit and palm. However, the activation distribution is slightly different between forward and backward orders. For the within-digit dimension, the results show a mirrored layout at the boundaries of Brodmann 3a, 3b, 1, and 2, i.e., distal (P1)-to-proxiam phalanx (P2) representation in area 3b but P2-to-P1 representation in area 1. This phenomenon is not as clear in all participants.

After extracting all the time-course data of each voxel that survived in phase-encoding analysis, we used Bayesian pRF to model the somatotopic map. Similar to the visual space, between- and within-digits are orthogonal. We defined a 2D hand space in the Cartesian coordinate system (Fig. 3*a*). We used a 2D Gaussian function to establish the models. After setting a second threshold at a posterior model probability >0.95, both the Gaussian and DoG models showed similar representations as the phase-encoding analysis (Fig. 3*b*).

Furthermore, we compared the model performance according to their negative variational free energy (Fig. 3*c*). We subtracted the F_Gaussian_ and F_DoG_ for each voxel to find the model with the most accurate and least complex explanation. Small voxels could be explained better by the Gaussian model (F_Gaussian_−F_DoG_ ≥ 3). Although some voxels had slightly larger F_DoG_ values (F_Gaussian_−F_DoG_ < −3, 11.966% in BA3a, 15.383% in BA3b, 13.179% in BA1, and 6.878% in BA2), most of the voxels were indeterminate between the 2 models (|F_Gaussian_−F_DoG_| < 3, 85.238% in BA3a, 81.806% in BA3b, 85.951% in BA1, and 92.584% in BA2).

### Activation Location and Relative Volume of Hand Representations

**Figure 4:**
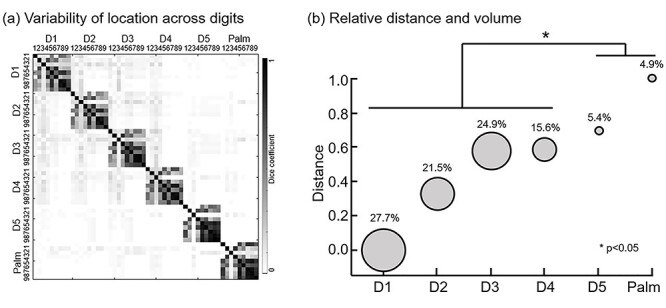
Location and relative volume of the somatotopic map. (*a*) Variability of pRF center location across participants and digits based on the Dice index. (*b*) Relative distances between D1-D2, D1-D3, D1-D4, D1-D5, and D1-Palm. Relative volumes are represented by the size of the colored circle. The colors correspond to each digit.

We only showed the results of the DoG model because they combined the forward and backward sessions and could explain the activation of most voxels. We compared all ROI pRF centers of somatotopic maps across participants and digits based on the Dice index. The index varied from 0 (no overlap, white) to 1 (perfect overlap, black) (Fig. 4*a*). For qualitative observation, dialog submatrices showed a higher Dice index, suggesting a high degree of intradigit overlap across participants. In addition, off-dialog submatrices with a lower Dice index indicated a low interdigit overlap degree. The quantitative index of MDR was 22.974. The permutation test (5000 samples) confirmed the significance of observing the Dice matrix pattern (*P* < 0.0001).

To quantify the location of each digit and palm, we regarded D1 as the coordinate origin and calculated the Euclidean distance of D1-D2, D1-D3, D1-D4, D1-D5, D1-Palm. Then, the results were normalized by the distance of D1-Palm. There was a clear increasing distance from D1 to Palm. In addition, D1 to D4 had a significantly higher relative volume than D5 to Palm (paired *t*-test, 5000 bootstrap samples, *P*_D1−D5_ = 0.001, *P*_D1−Palm_ = 0.001, *P*_D2−D5_ = 0.019, *P*_D2−Palm_ = 0.011, *P*_D3−D5_ = 0.001, *P*_D3−Palm_ = 0.003, *P*_D4−D5_ = 0.011, *P*_D4−Palm_ = 0.024).

### pRF Configurations in Hand Space

**Figure 5:**
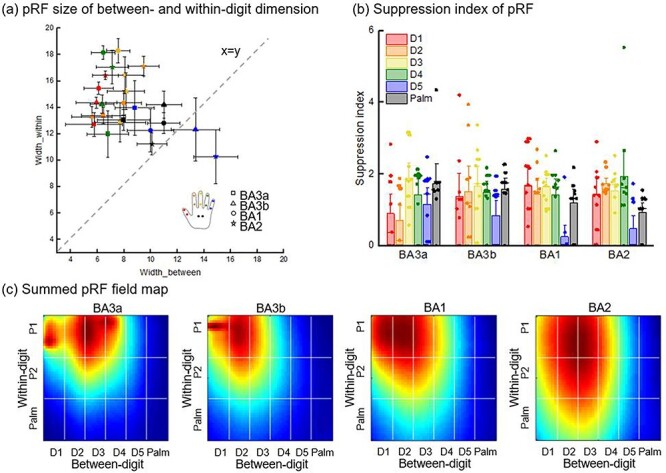
The pRF configurations in each ROI. (*a*) The pRF sizes of between- and within-digit dimensions. The line *x* = *y* means that the widths of the 2 dimensions are equal; in other words, the shape of the pRF is a circle. (*b*) Suppression index of pRF. The color corresponds to each digit. The scatter represents each participant. (*c*) Summed pRF field maps across voxels, which represent the primary center position and the spread distribution of pRF.

For each ROI, we investigated the pRF characteristics. First, the pRF width of the between- and within-digit dimensions was averaged for each digit across participants (Fig. 5*a*). If the point falls on the line *x* = *y*, the pRF is a circle. The more deviated from this line, the greater the pRF ellipse. The results showed that the pRFs of D5 and Palm were closer to this line, meaning that they were more rounded. In addition, the suppression index was calculated to indicate the center-surround configuration. D5 had a significantly smaller suppression index than the other digits in BA3a (paired *t*-test, 5000 bootstrap samples, *P*_D3−D5_ = 0.024), BA2 (*P*_D2−D5_ = 0.029, *P*_D3−D5_ = 0.014), and especially in BA1 (*P*_D1−D5_ = 0.014, *P*_D2−D5_ = 0.034, *P*_D3−D5_ = 0.004, *P*_D4−D5_ = 0.044, *P*_D5−Palm_ = 0.025). In addition, D1 and D2 had smaller suppression indices in BA3a (*P*_D1−D3_ = 0.047, *P*_D1−Palm_ = 0.026, *P*_D2−D3_ = 0.041). There was no significant difference across digits in BA3b (*P* > 0.05). We next visualized the somatosensory pRF field maps across voxels (Fig. 5*c*).

### Correlation of pRF Size and Activation Volume

**Figure 6:**
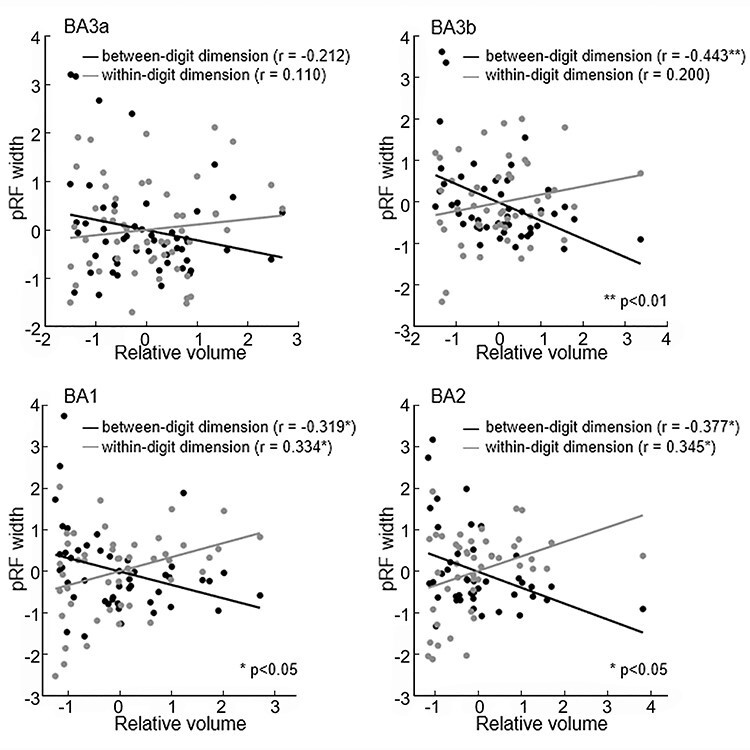
Relationship between relative activation volume and pRF width in each ROI. Black dots and lines represent the results of the Pearson correlation between the relative volume and pRF width of the between-digit dimension. Red dots and lines represent the results of the Pearson correlation between the relative volume and pRF width of the within-digit dimension.

The relative activation volume and pRF width of the between-digit dimension showed significant negative correlations in BA3b (*r* = −0.443, *P* = 0.002), BA1 (*r* = −0.319, *P* = 0.029), and BA2 (*r* = −0.377, *P* = 0.012) (Fig. 6). The greater the activation area, the smaller the pRF was. However, there was a significant positive correlation between the relative activation volume and the pRF width of the within-digit dimension in BA1 (*r* = 0.334, *P* = 0.022) and BA2 (*r* = 0.345, *P* = 0.022) (Fig. 6).

## Discussion

Along the tactile ascending pathway, somatotopy is an important guiding principle for sensory fiber organization. In this study, we used high-resolution fMRI at 7 T and focused on the somatotopic map of the left hand in right-handed participants. We defined a 2D hand space with between- (D1-D2-D3-D4-D5-Palm) and within-digit dimensions (P1-P2-Palm). Bayesian Gaussian and DoG ellipse models were established to explore the pRF characteristics in 2 dimensions. First, we investigated the activation location and extent area of each digit in S1. In addition, we divided S1 into 4 subareas (BA3a, BA3b, BA1, and BA2) according to probabilistic maps and functional activation. The pRF configurations along 2 dimensions were recorded in each voxel and averaged in each ROI, which were visualized in a summed coverage field map. The current results contribute to the general somatotopic organization and pRF characteristics of the left hand in the right hemisphere.

In the between-digit dimension, the results of both phase encoding and Bayesian pRF analysis showed orderly representation organized along D1-D2-D3-D4-D5-Palm in the postcentral gyrus. The thumb is most inferior and lateral, and other digits are represented at increasingly superior and medial locations, followed by the palm (Figs. 2 and 3). This is consistent with previous studies on the right hand (Schweizer et al. 2008; Schweisfurth et al. 2014). Studies that stimulated 5 proximal phalanges of the left hand found similar somatotopic organization in the right hemisphere (Sanchez-Panchuelo et al. 2010; Stringer et al. 2014). The representation of left- and right-hand were arranged in a similar order in individual brain or normalization space (Pfannmoller et al. 2016). In the within-digit dimension, we found mirrored patterns at the areal boundaries of area BAs (i.e., the proximal-to-distal phalanx representation is posterior to anterior in BA 3b but anterior to posterior in BA 1). Studies on nonhuman primates using electrophysiology have reported similar multiple representations of within-digit maps in S1 (Kaas et al. 1979). Schweisfurth et al. (2014) investigated the within-digit map of D1 to D5 and reported that only D4 and D5 showed orthogonal orderly patterns in BA 3b (Schweisfurth et al. 2014). However, some studies did not find such a consistent within-digit somatotopic map (Overduin and Servos 2004, 2008; Schweisfurth et al. 2011).

In the phase-encoding analysis, the results of the forward and backward stimulation directions were slightly different, which may be due to delays in the hemodynamic response (Sanchez-Panchuelo et al. 2010). The pRF model-based method is well suited to more types of experimental design, which could eliminate the difference between the 2 directions (Dumoulin and Wandell 2008). Moreover, the Bayesian pRF method could account for variability in the hemodynamic response across the brain. After considering the interaction between stimulus and pRF size, the mirrored pattern of the within-digit map became blurred. Traditional phase-encoding analysis is based on the “winner-takes-all” strategy, and the activation location of each phalanx may deviate due to the larger pRF width along the within-digit dimension (Fig. 5*a*). We used both Gaussian and DoG models to perform the estimation. Most voxels are indeterminate between the 2 models, whereas some voxels could be explained slightly better by the DoG model. This suggested that some neurons in S1 have center-surround properties with both excitation and inhibition regions (DiCarlo and Johnson 2000; Kuehn and Pleger 2020).

Although a previous study reported the intraparticipant reproducibility and interparticipant variability of the right-hand somatotopic map (Kolasinski et al. 2016a), our results showed low variability of location across participants based on the Dice index, as expected (Fig. 4*a*). A study using a motor task found that natural hand use shapes the relative arrangement of finger-specific activity patterns in the sensory-motor cortex (Ejaz et al. 2015). Researchers compared the somatotopic map of D3 and D5 for both hands of right-handed participants and reported that the left hand showed a more consistent presentation across participants than the right hand (Schweisfurth et al. 2015). From this point of view, studying the reorganization of the nondominant hand may be more reliable in patients because it could better rule out individual differences and focus on changes brought by disease. In addition, there is a slight decrease in off-dialog submatrices from top left to bottom right, which suggests that there is overlap between adjacent digits, which decreases with increasing digit distance (Besle et al. 2014). In addition, our results showed a significantly larger relative volume of D1-D4 than D5-Palm (Fig. 4*b*). We did not observe a larger representation for thumb, which is different from the right-hand results (Martuzzi et al. 2014). Thumb magnification was also not found in nonhuman primates (Kaas 1983). The larger volume of the right thumb may be related to more haptic explorations, which is a potential sign of evolution (Hashimoto et al. 2013). In addition, the participants included in our study were more female whereas Martuzzi et al. only investigated 10 males (Martuzzi et al. 2014). Gender may also be a major influence. Several studies investigated the differences of somatotopic map related to gender, such as breast (Di Noto et al. 2013; Beugels et al. 2020). Differences in other parts of the body are worth exploring in future studies.

For pRF configurations, we measured the pRF width in between- and within-digit dimensions. The results showed an elliptical shape of pRF in S1, in which the long axis was along the within-digit dimension. This was consistent with a previous study that reported different spatial orientation sensitivities of neurons in S1 (DiCarlo and Johnson 2000). The ellipse aspect ratio was different across digits: the pRF of D5 and Palm tended to be more rounded, whereas other digits were more elliptical (Fig. 5*a*). This indicates that the voxels corresponding to D1, D2, D3, and D4 are more concentrated in the information processing within a specific digit. The functional differentiations of voxels with D5 and Palm pRF centers are not as obvious, so they have large pRF sizes and responses to more digits. The summed somatosensory pRF field map showed that the pRF size was larger in BA1 and BA2 than in BA3a and BA3b. This is in line with a previous study based on the surface vertex (Puckett et al. 2020), and a study showed less overlap between adjacent digits in the posterior bank of the central sulcus than in the postcentral gyrus (Besle et al. 2014). Nonhuman primate studies also reported similar results in which neurons in BA1 and BA2 responded to more digits (Sur et al. 1985; Sripati et al. 2006; Ashaber et al. 2014). Our results confirmed the hierarchical processing in S1 that BA3b and BA1 processed signals from skin, and BA2 combined the signals (Costanzo and Gardner 1980; Gardner 1988). In addition, we found that the suppression index of D5 was significantly smaller than that of other digits, especially in BA2. A similar reduction was observed in the visual cortex, which should decrease the suppression index with eccentricity (Zuiderbaan et al. 2012). As the pRF was estimated from a population of neurons in a voxel, increasing position variance leads to a decrease in the center-surround configuration of the pRF (Zuiderbaan et al. 2012).

Furthermore, we found a negative correlation between the relative volume and pRF width in the between-digit dimension (Fig. 6, black line). This is supported by a previous study showing that cortical activation is highly related to tactile spatial acuity (Duncan and Boynton 2007). There was a decrease in tactile spatial acuity from the thumb to the little finger (e.g., increased pRF size in the between-digit dimension), which was reflected in the decreased relative volume activated in S1. However, in the within-digit dimension, the results showed a positive correlation between the relative volume and pRF width (Fig. 6, red line). One explanation is that when estimating the pRF of the within-digit dimension, all 5 proximal or distal phalanges were stimulated together. Attention was allocated to each digit, which may cause uneven distribution across digits, such as paying more attention to D1 and less attention to D5. Previous studies reported attention-induced changes in pRF in the visual cortex (Klein et al. 2014). Attention was able to elicit orderly gradients in somatotopic maps even though there was no tactile stimulus (Puckett et al. 2017). Researching the interaction between attention and somatotopic processing of digits is important to understand the neural basis of S1 (Kida et al. 2018). Furthermore, the response of the cortical column from different afferent fibers of digits may be influenced by lateral inhibition in the synchronous condition (Kuroki and Nishida 2018). The other explanation is that the mechanisms of between- and within-digit representation were separate. A plasticity study revealed the “boundary-adjacent” feature in the between-digit dimension and continuity in the within-digit dimension (Grajski 2016). When manipulating objects in a wrapped posture, the probability of the whole digit touching the surface of the object decreased from D1 to D5. This means that the within-digit synergy gradually decreases from D1 to D5. This may cause a decrease in pRF from D1 to D5 in the within-digit dimension, especially in BA2, where signals are integrated together.

There are some limitations in our study. First, we only focused on the somatotopic map of the human hand in S1 and defined the hand space in arbitrary units. It is possible that numerous neuronal populations responded to other body parts. This could be improved to extend the experiment to a whole-body somatotopic map to further examine the pRF characteristics in S1. In addition, we used discrete points of tactile stimulus to induce the somatotopic map due to the limitation of scanning time, which could be improved by using more intensive continuous stimuli to explore fine-grained characteristics of pRF. Second, we only investigated the activation induced by low-frequency tactile stimuli. There are 4 types of mechanoreceptors in human glabrous skin that optimally respond to different tactile stimuli. Our results may reflect their mixed performance. Designing more complex stimulations could help us understand specific pRFs corresponding to each type of mechanoreceptor, such as using different frequencies or shapes.

In conclusion, we showed the somatotopic organization of the left hand in right-handed participants. The left-hand somatotopic map was organized in a certain order from thumb to palm, which was similar to that of the right hand. However, our results showed low variability of left-hand representation across participants, which may serve as a marker for disease-related reorganization. The Bayesian pRF method has higher robustness than phase-encoding analysis, which is suitable for various experimental designs. For the pRF configurations, D1, D2, D3, and D4 showed elliptical shapes, and the long axis was along the within-digit dimension. This was related to the degree of functional differentiation across digits. In addition, the results showed different change trends of pRF width in the between- and within-digit dimensions. The current findings offer new possibilities for neuroscientific investigation of somatosensory information processing.

## Funding

National Key R&D Program of China (grant number 2018YFC0115400), the National Natural Science Foundation of China (grant numbers 61727807, U20A20191, 82071912), the Beijing Municipal Science and Technology Commission (grant numbers Z191100010618004).

## Notes

We acknowledge and thank the participants involved in this study. *Conflict of Interest:* The authors declare that the research was conducted in the absence of any commercial or financial relationships that could be construed as potential conflicts of interest.

## Abbreviations

population receptive field (pRF), primary somatosensory cortex (S1), difference of Gaussian (DoG), region of interest (ROI).
